# Clinical Trial Safety Surveillance in Africa: Experts’ Perspectives on Current Practices and Opportunities

**DOI:** 10.3390/vaccines13111139

**Published:** 2025-11-05

**Authors:** Chioma S. Ejekam, Kwasi A. Nyarko, Onome T. Abiri, Yakubu N. Beno, Rhanda M. Adechina Adehan

**Affiliations:** 1 African Vaccine Regulatory Forum, Disease Prevention and Control Cluster, World Health Organization Regional Office for Africa, Brazzaville P.O. Box 06, Congo; nyarkok@who.int (K.A.N.); rhanda.adechina@who.int (R.M.A.A.); 2 Pharmacovigilance and Clinical Trial Department, Pharmacy Board of Sierra Leone, Central Medical Stores Compound New England Ville, Freetown P.O. Box 322, Sierra Leone; anome.abiri@usl.edu.sl; 3 Department of Pharmacology and Therapeutics, College of Medicine and Allied Health Sciences, University of Sierra Leone, Jomo Kenyatta Rd, Freetown P.O. Box 84, Sierra Leone; 4 Clinical Trials, Biologics and Vaccine Division, Drug Evaluation and Research (DER) Directorate, National Agency for Food and Drug Administration Control, Plot 1, Isolo Industrial Area, Oshodi-Apapa Expressway, Isolo 102215, Nigeria; beno.y@nafdac.gov.ng

**Keywords:** clinical trial, drug safety monitoring system, safety surveillance systems, pre-licensure safety surveillance, pharmacovigilance, Africa

## Abstract

Clinical trial (CT) safety surveillance is critical to protecting participants and ensuring reliable evidence on the safety and efficacy of new medical products. This is especially relevant in Africa, where CT activity remains limited, regulatory maturity varies, and drug safety surveillance systems are under-resourced despite considerable demographic advantages and genetic and cultural diversity. Pre-licensure safety monitoring is a vital yet underdeveloped element of the research ecosystem, and the absence of a regional repository for safety data constrains early detection of risks, particularly in multi-country trials. To assess the current state of CT safety surveillance in Africa, a landscape analysis of the systems for clinical trial safety data reporting, collation, and analysis was conducted. Expert perspectives were synthesized to describe existing practices, identify key gaps, and propose opportunities for strengthening systems. Findings revealed limited regulatory capacity, limited drug safety monitoring expertise, and inadequate resources for causality assessment and aggregate data analysis. Despite these challenges, opportunities exist to strengthen CT safety surveillance through digitization of reporting systems, harmonization of serious adverse event forms, regional collaboration, and capacity building for strengthening the ecosystem. Experts emphasized the need for collaboration among regulators of member states, availability of electronic CT management platform in member states and a regional pre-licensure safety data repository to enable timely evidence generation, support member states, and ensure appropriate linkages between pre-licensure and post-market surveillance. Strengthening CT safety surveillance is critical for safeguarding participants, promoting ethical research, and enhancing Africa’s role in global clinical research. The results of this landscape analysis provide a roadmap for building a coordinated model for pre-licensure safety monitoring across the continent.

## 1. Background

Clinical trials (CTs) are fundamental to the development of new medical interventions, advancing medical knowledge, improving treatment outcomes, and establishing the safety and efficacy of new medical products [1,2]. Integral to this is the continuous monitoring of safety across the life cycle of medical products from design, pre-licensure phases, and clinical trials to post-licensure use and environmental disposal, to ensure that risks to participants and end-users are identified, assessed, and mitigated, which is crucial for developing effective and safe treatments [3,4]. Pre-licensure safety surveillance—the monitoring of adverse events during the clinical development of a product—plays a particularly critical role in protecting participants, establishing the risk–benefit profile of investigational products, and informing post-licensure safety strategies [5,6]. As investigational products transition from the controlled trial environments to broader, more diverse populations in real-world settings, robust safety surveillance becomes even more important. Prelicensure safety systems complement post-market pharmacovigilance by identifying early safety signals that might otherwise go undetected in routine clinical settings [7]. In low- and middle-income countries (LMICs) and data-limited settings, such as in vulnerable populations, particularly in Africa, the need for effective pre-licensure surveillance is amplified by several contextual factors. These include low clinical trial infrastructure, underdeveloped regulatory authorities, and the absence of a centralized or regional clinical trial safety database similar to Eudravigilance, the European Union’s system for monitoring the safety of medicines and clinical trials [8]. Moreover, Africa’s significant genetic diversity, especially in pharmacogenetically relevant genes such as cytochrome P450, suggests that drug responses may differ markedly across populations [9]. Despite this, Africa remains underrepresented in global clinical research, and its populations are often treated as a uniform entity in drug development programs. This underrepresentation, combined with known pharmacogenomic variability, raises the risk of unanticipated adverse reactions when globally approved products are introduced to African populations.

Although international guidelines such as those from the Council for International Organizations of Medical Sciences (CIOMS) and the International Council for Harmonisation of Technical Requirements for Pharmaceutical for Human use (ICH) emphasize the need for standardized safety monitoring, implementation across African countries remains inconsistent [3,10]. Few African countries have dedicated pre-licensure safety surveillance systems, and those that do often operate in silos, lacking coordination and data-sharing mechanisms that are essential for timely safety signal detection, especially in multi-country trials.

Currently, no centralized or regional repository exists for clinical trial safety data across Africa. Individual countries manage adverse event reports independently with varying levels of regulatory capacity and maturity. This fragmentation hinders effective monitoring and undermines efforts to harmonize regulatory practices across the continent. Furthermore, investigators in Africa are often required to report adverse events to multiple bodies—NRA, National Ethics Committees, and sponsors without standardized tools—which may lead to delays and limited data comparability [6,11].

To address these gaps, a coordinated framework for pre-licensure safety surveillance is urgently needed. Such a framework should include standardized data collection tools and procedures, efficient mechanisms for coordination and collation, and analysis of safety data, signal detection, and transparent channels for stakeholder communication [7,9,10].

**Figure 1:**
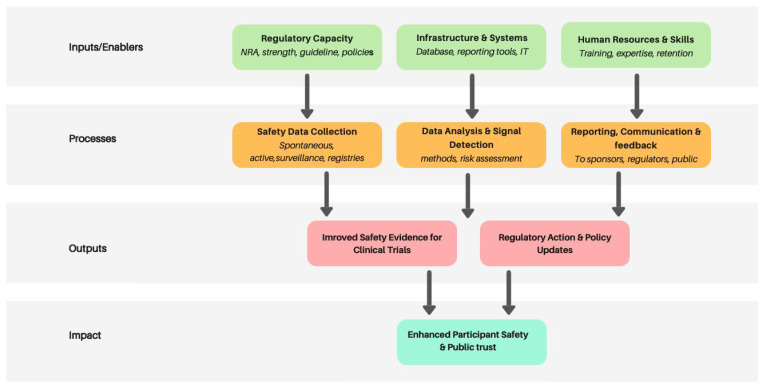
Conceptual framework for clinical trial safety surveillance.

Key actors—investigators, sponsors, ethics committees and National Regulatory Authorities (NRAs)—must be empowered with clear responsibilities and supported by systems that facilitate collaboration and information sharing [1,6,7]. Beyond infrastructure, building human resource capacity and promoting regulatory harmonization will be essential to support sustainable surveillance systems across the continent. A conceptual framework illustrates this continuum: from enabling inputs (e.g., regulatory capacity, infrastructure, and trained personnel) as the foundation, through core processes of safety management activities such as safety data collection, analysis, and communication, to outputs like regulatory action and safety evidence, all contributing to improved participant protection and informed decision-making (Figure 1).

One important initiative in this space is the African Vaccine Regulatory Forum (AVAREF), a network of NRAs and NECs from Africa’s member states, with its secretariat coordinated by the World Health Organization (WHO) nearly two decades ago. AVAREF has played a pivotal role in strengthening clinical trial oversight and facilitating joint reviews for multi-country clinical trials. Its efforts have supported research in high-priority areas including Ebola, malaria, tuberculosis, COVID-19, monkeypox, and Lassa fever [12].

As the clinical trial Technical Coordinating Committee of the African Medicines Regulatory Harmonization (AMRH) initiative, AVAREF has laid important groundwork for the operationalization of the African Medicines Agency. Its adoption of a life-cycle approach to product safety integrates both pre-licensure monitoring and post-authorization safety activities. AVAREF has also developed Good Clinical Practice (GCP) tools—checklists and reporting templates—endorsed by NRA leadership to promote ethical and scientific rigor in trials [13].

To assess the state of clinical trial safety surveillance in Africa, AVAREF conducted a landscape analysis of safety data reporting, collation, and analytical practices. This paper presents findings from expert perspectives, outlines current practices, identifies key gaps, and provides evidence-informed recommendations to strengthen prelicensure safety surveillance systems across the continent.

## 2. Approach

To understand the state of clinical trial safety surveillance among the 47 member states within the WHO African Region, the AVAREF secretariat sent out an electronic survey to clinical trial focal points within the NRAs who were already part of the AVAREF network and participating in the secretariat activities. Twenty of the 47 clinical trial focal points from member states responded. The survey assessed the presence of guidelines, procedures, policies, and regulations for clinical trials, as well as systems for adverse event (AE) reporting, storage, signal generation, and signal management within NRAs. The survey was deployed in English and French.

Following the survey, two cohorts of virtual meetings were convened to gain a deeper understanding of the CT safety surveillance landscape and to further explore national experiences and participants’ perspectives. These meetings involved CT focal points from the 20 countries who had responded to the electronic survey, and an expert from an African CT network, to ensure diverse viewpoints. The virtual meeting was coordinated by a CT safety focal point at the AVAREF secretariat. The discussions centered on key aspects of adverse events reporting in clinical trials, spanning the entire process from data reporting to signal management, as well as the feasibility of a continental CT safety repository. The discussions were guided by structured questions (Table A1) and conducted in English and French. The respondents were provided with the discussion questions prior to the meeting.

As agreed during the consultations, the views expressed reflect the perspectives of individual focal points as experts, not official country positions; therefore, the names of responding countries are not disclosed.

## 3. Results

### 3.1. Safety Surveillance System for Clinical Trial in Africa

#### 3.1.1. Findings from the Electronic Survey

The electronic survey revealed that 17 (85.0%) of the 20 responding countries had ongoing clinical trials. Among these, 14 countries (70.0%) reported having safety surveillance systems in place for coordinating, reporting, and collating pharmacovigilance and clinical trial safety data. Of these, 10 countries (71.4%) confirmed having both clinical trial safety monitoring and post-market surveillance systems within the NRA but collated and stored in different databases. Interestingly, only 10 of the 20 countries (50.0%) reported having policy documents within their regulatory authority that provided essential guidance for clinical trial safety surveillance. Similarly, half of the countries (50.0%) had adopted GCP guidelines, with all of them adopting the AVAREF clinical trial inspection guideline, checklists and templates.

Specific legal provisions supporting clinical trial safety monitoring were present in 12 (60.0%) of the countries. These provisions included the authority to request information, ensure privacy, and maintain confidentiality. Furthermore, 13 countries (65.0%) reported having legal requirements for investigators or sponsors to mandatorily report all suspected AEs to the NRA. Among these, 12 countries (92.3%) required marketing authorization holders or sponsors to conduct similar safety surveillance activities for investigational new drugs as those required by competent regulatory authorities in other areas.

For the reporting of serious adverse events (SAEs) during clinical trials, 13 countries (65.0%) utilized paper forms, electronic forms, or a combination of both. Of these, 12 countries (92.3%) used paper forms that were emailed to a designated address within the regulatory authority, while 7 countries (53.8%) employed electronic data collection tools, and 6 countries (46.1%) utilized both paper and electronic systems.

**Figure 2:**
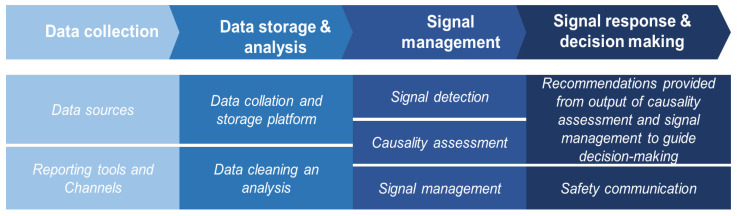
Process schematic for clinical trial safety surveillance system.

Regarding safety signal generation and management, only 7 countries (35.0%) reported having a system for managing safety signals during clinical trials. Among these, safety report analysis, including causality assessment, signal detection, and signal management, was conducted by personnel within the NRA, such as clinical trial teams, pharmacovigilance officers, or technical advisory committees. Of the seven countries with systems for managing safety signals, six (85.7%) indicated that their responsible staff had been trained in signal generation and management. The survey findings provided critical insight into the existing infrastructure and shaped the discussion points addressed during the contextual deep dive in the virtual meeting. Figure 2 shows a typical process schematic for a clinical trial safety surveillance system. It illustrates a simplified clinical trial safety surveillance system beginning with data collection from potential safety data sources through designated reporting tools and channels. The collected data is then collated and stored, cleaned, and analyzed in a database. Signal detection is performed through data mining and causality assessment to identify potential safety concerns. The outcomes of signal management inform the development of recommendations, which are communicated to relevant stakeholders to inform evidence-based decision-making.

#### 3.1.2. Findings from the Virtual Meetings

*CT Safety Data Reporting and Custodianship*. Discussions revealed that, across the AFRO region, the responsibility for reporting adverse events during clinical trials rests with both the trial sponsor and the principal investigator (PI). It is mandatory for the PI, the clinical research organization (CRO), or the sponsor to report safety data to the NRA, which acts as the custodian of CT safety data. In some countries, the PI is also required to report safety data to ethics committees, such as national ethics committees, institutional review boards, or regional ethics committees.

Safety reporting typically flows from the PI to the sponsor and then to the NRA, with some countries requiring submissions to both the NEC and the NRA. Although CT participants rarely report safety concerns, in rare instances, participants may notify the NRA directly if they suspect malpractice. The CIOMS VI Report recommends that all adverse events, whether serious or non-serious, should be documented during the development of any clinical trial, regardless of the investigator’s or sponsor’s presumed relationship to the study agent. This comprehensive collection enables the subsequent assessment of causality using standardized methods for both individual cases and aggregate data.

Participants shared that NRAs typically receive serious adverse events (SAEs) within 7 days and suspected unexpected serious adverse reactions (SUSARs) within 24–48 h. All other adverse events are submitted as line lists on a monthly, quarterly, biannual, or annual basis, depending on the member state. Some countries only receive SUSARs, while others expect all SAEs and SUSARs. To address delayed submissions from foreign sponsors, member states often bestow on the PIs the responsibility to ensure the timely submission of SAEs.

Foreign trial sites in multi-country clinical trials are mandated in some countries to submit safety reports for investigational new drugs (INDs) within specific timelines, including SAEs and SUSARs. Participants noted that managing safety data is easier with local sponsors, as foreign sponsors often delay submissions due to conducting causality assessments before sharing data with NRAs or do not submit at all. While safety data from foreign sites are typically limited to SUSARs with periodic line-list submissions, regulatory authorities require that data from all participating countries be shared, as identified during the clinical trial application process. Challenges remain in receiving these and integrating safety data from foreign trial sites, despite mandatory reporting requirements.

*Reporting Format and Channels*. Most member states have adopted the CIOMS SAE format for reporting SAEs, in alignment with the ICH E2B R3 standards. While both paper and electronic SAE forms are available, only a few countries use electronic reporting portals. Sponsors and investigators submit safety data—SAEs and SUSARs to the NRAs and/or NECs through these portals, or via email for countries using paper forms. For those with electronic platforms, safety reports are directly uploaded into the database. In cases where reports are sent in PDF format for countries that use the paper forms, a responsible person in the NRAs manually enters the data into the system, creating potential delays.

Some pharmaceutical companies convert their reports into XML format, making it easier for NRAs to integrate the data into Vigiflow, the database used for post-marketing safety data in most countries and also used to store CT safety data in many African countries.

However, many countries maintain separate pharmacovigilance and CT safety databases, typically within their Directorate (Division/Unit) of Product Safety. Access to the CT database is restricted even within the CT unit, and only designated personnel are permitted to manage or review the data.

*CT Safety Data Collation and Storage*. Two prevalent approaches to CT safety data collation and storage were identified. Most of the countries within the region rely on Vigiflow for local storage of their CT safety data but do not upload the data to the global Vigibase system. Often, a designated officer receives the safety report and feeds it into the Vigiflow database. There are 2 pathways for sharing CT safety data—via the XML format that will be coded and shared directly or via the manual entry of the pdf documents. Safety data is either manually entered from PDFs or uploaded as XML files and shared directly by sponsors. Delays in manual data entry due to large PDF backlogs were a recurring issue. Countries like Kenya, Nigeria, South Africa, and Uganda utilize electronic platforms to manage CT applications and store CT safety data. For instance, Nigeria’s Electronic Clinical Trial Application Platform (ECTAP) includes modules for SAE submission and final Good Clinical Practice inspection reports.

*Causality Assessment and Signal Management*. Reporting of adverse events during clinical trials could generate safety signals, which, according to the European Medicines Agency, is the information on a new or known AE that is potentially caused by a medicine and that warrants further investigations.

Participants revealed that causality assessments are typically conducted only for SAEs and SUSARs, while non-serious AEs are line-listed. Causality assessment could be based on analysis of multiple cases/aggregate data (quantitative assessment) or assessment of individual adverse events by the investigator (qualitative) using the investigator’s brochure as a reference, and clinical review is also done. The causality assessment output is a report on the relatedness of the event to the study treatment or intervention, with various gradients of this ‘relatedness’ used. The discussion showed that aggregate analysis of multiple cases is rare, as most countries lack the capacity for such assessments. According to the focal points, the NRAs often rely on individual assessments conducted by a designated officer or causality assessment team using standardized tools. Outcomes are reviewed by technical or safety advisory committees (made of experts in the specific field of CT), which provide recommendations for further action, such as updating the investigator’s brochure or revising the severity grading. However, the discussions showed that most of the NRAs often do not have an established Technical Advisory committee for CTs but often tend to engage different expert sub-committees based on the CTs and safety issues, who then assess the reports of the causality assessment, and they inform the NRA on the appropriate actions. Some participants shared experiences where NRAs conducted their own causality assessments and arrived at different conclusions from those of the sponsors. In several instances, sponsors initially classified events as “unrelated,” but the NRAs’ assessments determined them to be “related.” These findings often prompted foreign sponsors to update the investigator’s brochure and revise the causality assessment report to reflect the event as “related” instead of “unrelated”.

To ensure confidentiality, access to CT safety data is restricted, even within CT safety units. Staff and advisory committee members sign declarations of confidentiality and conflict of interest before reviewing or managing safety data. Data protection measures include de-identification and coding of reports, and databases are accessible only to designated personnel.

### 3.2. Challenges Identified with Clinical Trial Safety Monitoring System in the African Region

The CT focal points shared their unique experiences and collectively identified the following as potential challenges to effective clinical trial safety monitoring within the region

Inadequate financial resources for clinical trials: The region faces a shortage of funding, infrastructure, human resources, and capacity-building opportunities for clinical trials, in addition to limited clinical trials ongoing on the continent despite the rich genetic diversity. Insufficient training and a lack of dedicated personnel further hinder the effective implementation of safety monitoring frameworks. This includes poor funding for safety monitoring, especially for CT in the NRAs, consequently affecting the establishment of an effective system in place for safety surveillance. This restricts the ability of regulatory bodies to effectively monitor and respond to safety concerns, leaving gaps in trial oversight. As noted from the virtual meeting, some of the countries store their CT safety data in the WHO Vigiflow, designed for local storage of pharmacovigilance data, while seeking funding support to develop a separate clinical trial electronic database. Additionally, the limited resources and expertise consequently affect the ability of the NRAs to have an established and functional Technical Advisory Committee for CT.

In many African countries, there is an unavailability of clinical trial guidelines and documents that provide clear protocols for conducting and monitoring clinical trials. This gap leads to inconsistencies in how safety is monitored and managed. With the availability of AVAREF’s comprehensive CT guidelines, countries have the option to adapt or adopt these guidelines.

Lack of harmonization of clinical trial tools and processes: Even within individual countries, clinical trial processes and tools are not harmonized. Some sponsors come with their own SAE forms for every CT. This lack of standardization creates inefficiencies and complicates the monitoring of trial safety. Countries are making an effort to harmonize SAE forms so that investigators, CROs and sponsors use the same SAE form for reporting CT safety data. It is important to understand that harmonization does not imply that countries adopt the same processes; rather, it ensures that at least there is consistency within the country and alignment with globally recommended minimum safety monitoring requirements to aid comparability in assessment. In addition, confusion often arises over safety reporting obligations. Investigators and sponsors have distinct responsibilities under ICH GCP E6 R3, while national regulations may impose additional requirements. This division of roles, combined with varying timelines across countries, may complicate harmonization and practical implementation in multi-country trials.

Limited capacity and infrastructure to handle clinical trial adverse event reports: There is a significant shortage of trained professionals within the Regulatory authorities with the expertise to carry out causality assessment and signal management, making it challenging to adequately process and evaluate adverse events and respond to safety signals arising from clinical trials in a timely manner. Hence, there is a need to build capacity for this critical component of evidence generation.

The use of different causality assessment tools across clinical trials and countries leads to discrepancies in determining the relatedness of adverse events to the investigational medicinal product. This inconsistency hinders the comparability of assessment outcomes of clinical trial safety data.

Many countries within the region lack a robust legislative framework that mandates safety surveillance in clinical trials. This regulatory vacuum undermines the ability to enforce safety standards effectively. Similarly, the absence of specific legal provisions for clinical trial oversight limits the regulatory authority’s role in ensuring compliance with safety protocols. The legal framework provides the requirements for investigators or sponsors to mandatorily report all AEs to the NRA and in a timely manner throughout the life cycle of a CT, similar to what is practised by the United States Food and Drug Administration and European Medicines Agency [8]. Similarly, the legal frameworks mandate marketing authorization holders or sponsors to conduct similar safety surveillance activities for investigational new drugs as those required by competent regulatory authorities in other areas.

Many of the countries report CT safety data in paper form and manually manage safety reports in a spreadsheet. There was high interest from the participants in digitalizing the CT application-to-safety monitoring process, enabling electronic safety reporting and data management. Lack of advanced information management systems for processing clinical trial applications and collecting safety information poses a logistical challenge, delaying rapid and timely reporting of safety data, making it difficult to track and analyze safety data effectively.

Adverse events from clinical trials are often underreported, and when reported, the quality of the information provided is frequently insufficient for proper analysis and response.

Many African countries lack a national clinical trial registry, making it difficult to track ongoing trials and ensure compliance with safety monitoring requirements.

Insufficient and ineffective collaboration between relevant stakeholders creates silos that inhibit effective safety monitoring and data sharing.

## 4. Discussion and Recommendations

Participants highlighted opportunities within the current state of clinical trial safety surveillance and proposed mechanisms to address persistent challenges. A central recommendation was the harmonization of SAE reporting forms to improve data quality and comparability across trials. While harmonization does not require identical tools, alignment with CIOMS SAE guidance, ensuring inclusion of minimum reporting variables, safety reporting obligations and compliance with the E2B R3 format would enable consistency and transferability of safety data [3].

Equally emphasized was the need for a continental prelicensure safety surveillance system to complement Africa’s post-market pharmacovigilance framework. A database that is regionally appropriate, owned and hosted in Africa. Digitization of CT safety surveillance remains a high priority for member states, with many countries having already sought support to establish electronic CT safety databases, though with limited success to date. An electronic CT platform that would integrate application approvals with safety monitoring was considered critical by the member states. An efficient information management system would streamline processes, supporting real-time monitoring and evidence-based decision-making by regulatory authorities. However, a central repository alone would not enhance product safety; its value lies in the effective analysis and interpretation of collected information to guide timely regulatory actions. To achieve impact, such systems must be supported by clear regulatory provisions, guidelines for the collection and analysis of data, well-trained staff to work on the data, and standardized procedures that align with international standards.

Causality assessment emerged as another priority. The presence of a safety signal does not imply causation, but necessitates systematic evaluation. In line with CIOMS VI recommendations, causality assessments are required for all adverse events [3]. Yet aggregate analysis of multiple cases is rare due to limited capacity, leaving most NRAs to rely on individual assessments by designated officers or causality assessment teams. While individual-level reviews can flag early detection of emerging safety concerns and the characterization of events, aggregate analyses provide stronger evidence for drug-event attribution [14]. Building capacity for advanced data analysis, supported by comprehensive training for regulatory staff and researchers, will strengthen oversight and improve Africa’s attractiveness for hosting clinical trials.

The development and regular updating of clinical trial guidelines are essential to address emerging challenges and technological advances, while ensuring alignment with global regulatory frameworks. AVAREF provides clinical trial guidance documents and templates that are continuously updated to reflect evolving best practices [15]. Equally important are legislative provisions that mandate compliance with good clinical practices and safety monitoring requirements. Such legal frameworks should ensure that investigational new drugs undergo the same level of safety surveillance as required by competent regulatory authorities elsewhere. In line with international standards, it is also critical to enforce mandatory reporting of safety data from all foreign sites conducting the same trial or from sites that have previously conducted the same trial [8]. Strengthened legal frameworks ultimately empower regulatory authorities to uphold rigorous safety standards.

Adequate financial resources are essential for strengthening clinical trial safety monitoring. Investment in pharmacovigilance, capacity building, and infrastructure development not only ensures sustainable progress but may also attract additional funding to support trials in the region [16]. Furthermore, Africa’s rich cultural and genetic diversity, which is currently underutilized, offers valuable insights for tailoring treatment regimens and reducing adverse drug reactions. Hence, financial resources are needed to explore this.

Although AVAREF continues to strengthen capacity and provide oversight for clinical trials across the region, expertise in this area remains limited. As highlighted during the virtual discussion, many NRAs lack established or functional technical or advisory committees for CTs. This gap is partly attributable to inadequate funding, since these committees require financial resources to convene and operate effectively. A Clinical Trial Technical Advisory Committee typically includes diverse stakeholders such as researchers, health authorities, regulatory agencies, ethics bodies, health technology assessment groups, healthcare practitioners, community representatives, and professional associations to ensure comprehensive oversight [3].

Finally, effective collaboration among investigators, sponsors, ethics committees, NRAs, and public health programs such as the Expanded Program on Immunization is vital. Strengthening stakeholder engagement, supported by functional technical advisory committees and consistent resource allocation, will foster data sharing, efficiency, interoperability, and joint problem-solving. Equipping stakeholders with the tools and expertise to manage and communicate safety data will close existing gaps and promote a culture of excellence in clinical trial safety oversight across Africa.

## 5. Strengths and Limitations

A major strength of this paper is that, within the limits of our literature search, it is the first to address pre-licensure safety surveillance systems in Africa. However, there are two possible limitations. The survey relied on self-reported information from regulatory authorities, which may not fully capture the actual state of clinical trial safety surveillance infrastructure. Respondents may have over- or under-reported capacity, practices, or system functionality, leading to reporting bias. Although 20 countries participated, which might be due to the limited availability of clinical trial safety surveillance structures and systems in many African countries, their participation may not reflect all countries or intra-country variations in clinical trial safety surveillance. The results should therefore be interpreted as indicative rather than exhaustive of the regional landscape.

## 6. Conclusions

The findings revealed substantial gaps in the infrastructure, legal frameworks and technical capacity for clinical trial safety surveillance within the region. Strengthening this system will require greater investment, targeted capacity building, harmonization of tools and practices, and stronger legislative frameworks to empower regulatory authorities.

Leveraging opportunities for regional coordination and data sharing mechanisms for pre-market safety data analysis is critical, as it provides continuity into the post-market period and enables more efficient and reliable monitoring during the introduction of new medicines. A regional pre-licensure safety surveillance repository would accelerate timely evidence generation, support all member states, and enhance Africa’s role in the global clinical trial ecosystem.

Africa’s rich cultural and genetic diversity remains a largely untapped asset for innovation in medical product development. Realizing this potential requires a bold call to action: investment in infrastructure and collaboration to establish a centralized model for pre-licensure safety data management. Doing so will not only safeguard participants but also contribute to greater public trust and regulatory confidence in clinical research across the African region.

## Abbreviations

The following abbreviations are used in this manuscript:

AMRH	African Medicines Regulatory Harmonization
AVAREF	African Vaccine Regulatory Forum
CIOMS	Council for International Organizations of Medical Sciences
CRO	Clinical Research Organization
CT	Clinical Trial
GCP	Good Clinical Practice
ICH	International Council for Harmonisation of Technical Requirements for Pharmaceuticals for Human Use
IRB	Institutional Review Board
LMIC	Low–middle-income Country
NEC	National Ethics Committee
NRA	National Regulatory Authority

## Appendix A

**Table A1:** The table shows the structured questions that guided the discussions during the virtual meeting.

Themes	Questions Asked
CT safety data reporting and custodianship	- Who is the authority/custodian for CT safety data? - What are the sources of CT safety data/Who reports CT safety data to the regulatory authority? - What type of CT safety data is reported—serious and non-serious adverse events, or SUSARs only? - What is the channel of reporting?
CT safety data collation, storage, and analysis	- Is there a repository for the collation and storage of CT safety data? - How is CT safety data managed?
Causality assessment, signal generation, and management	- Is causality assessment or severity grading performed on CT safety reports? - Who performs the assessment? - How is signal detection carried out, and what actions are taken following signal detection and validation?
